# LLIN Evaluation in Uganda Project (LLINEUP): factors associated with ownership and use of long-lasting insecticidal nets in Uganda: a cross-sectional survey of 48 districts

**DOI:** 10.1186/s12936-018-2571-3

**Published:** 2018-11-13

**Authors:** Samuel Gonahasa, Catherine Maiteki-Sebuguzi, Sheila Rugnao, Grant Dorsey, Jimmy Opigo, Adoke Yeka, Agaba Katureebe, Mary Kyohere, Amy Lynd, Janet Hemingway, Martin Donnelly, Moses R. Kamya, Sarah G. Staedke

**Affiliations:** 1 Infectious Diseases Research Collaboration, 2C Nakasero Hill Road, Kampala, Uganda; 2 National Malaria Control Programme, Uganda Ministry of Health, Kampala, Uganda; 3 University of California, San Francisco, San Francisco, CA 94110 USA; 4 Liverpool School of Tropical Medicine, Pembroke Place, Liverpool, L3 5QA UK; 5 London School of Hygiene & Tropical Medicine, Keppel Street, London, WC1E 7HT UK

**Keywords:** Malaria, Long-lasting insecticidal nets (LLINs), Uganda, Vector control

## Abstract

**Background:**

Long-lasting insecticidal nets (LLINs) are a key malaria control intervention. To investigate factors associated with ownership and use of LLINs in Uganda, a cross-sectional community survey was conducted in March–June 2017, approximately 3 years after a national Universal Coverage Campaign (UCC).

**Methods:**

Households from 104 clusters (health sub-districts) in 48 districts were randomly selected using two-staged cluster sampling; 50 households were enrolled per cluster. Outcomes were household ownership of LLINs (at least one LLIN), adequate LLIN coverage (at least one LLIN per 2 residents), and use of LLINs (resident slept under a LLIN the previous night). Associations between variables of interest and outcomes were made using multivariate logistic regression.

**Results:**

In total, 5196 households, with 29,627 residents and 6980 bed-nets, were included in the analysis. Overall, 65.0% of households owned at least one LLIN (down from 94% in 2014). In the adjusted analysis, factors most strongly associated with LLIN ownership were living in a wealthier household (highest tercile vs lowest; adjusted odds ratio [aOR] 1.94, 95% CI 1.66–2.28, p < 0.001) and time since the last UCC (29–37 vs 42–53 months; aOR 1.91, 95% CI 1.60–2.28, p < 0.001). Only 17.9% of households had adequate LLIN coverage (down from 65% in 2014). Factors most strongly associated with adequate coverage were fewer residents (2–4 vs ≥ 7; aOR 6.52, 95% CI 5.13–8.29, p < 0.001), living in a wealthier household (highest tercile vs lowest; aOR: 2,32, 95% CI 1.88–2.85, p < 0.001) and time since the last UCC (29–37 vs 42–53 months; aOR 2.13, 95% CI 1.61–2.81, p < 0.001). Only 39.5% of residents used a LLIN the previous night. Age was strongly associated with LLIN use, as were household wealth and time since the last UCC. Children < 5 years (44.7%) and residents > 15 years (44.1%) were more likely to use nets than children aged 5–15 years (30.7%; < 5 years: aOR 1.71, 95% CI 1.62–1.81, p < 0.001; > 15 years: aOR 1.37, 95% CI 1.29–1.45, p < 0.001).

**Conclusions:**

Long-lasting insecticidal net ownership and coverage have reduced markedly in Uganda since the last net distribution campaign in 2013/14. Houses with many residents, poorer households, and school-aged children should be targeted to improve LLIN coverage and use.

*Trial registration* This study is registered with ISRCTN (17516395)

## Background

Over the past decade, impressive reductions in malaria cases and deaths have been documented worldwide, following substantial investment in malaria control [1]. Between 2000 and 2015, an estimated 663 million clinical cases of malaria were averted by malaria control interventions; nearly 70% of these were attributed to use of long-lasting insecticidal nets (LLINs) [2]. However, malaria control achievements have not been uniform, and recent evidence suggests that the global decline in malaria burden may have stalled [1]. In Uganda, expanded coverage of malaria control interventions has been associated with reduced malaria incidence and prevalence [3–5], but malaria control gains have been fragile in high transmission areas and the burden of malaria in Uganda remains high [1, 6, 7].

Long-lasting insecticidal nets have been shown to reduce malaria morbidity and mortality across a range of epidemiological settings, and are the most widely used vector control tool [8, 9]. To achieve and maintain universal coverage with LLINs, defined as universal access to and use of LLINs by populations at risk of malaria, the World Health Organization (WHO) recommends the distribution of one LLIN for every two persons at risk of malaria through mass campaigns, conducted every 3 years [10]. Between May 2013 and August 2014, Uganda conducted its first Universal Coverage Campaign (UCC), to distribute LLINs free-of-charge nationwide. The aim of the UCC was to deliver at least one LLIN for every 2 residents to over 90% of households [11]. This campaign was led by a national coordination committee chaired by the Uganda Ministry of Health supported by key implementing partners. LLINs were distributed locally by village health teams (VHTs). Uganda’s last Malaria Indicator Survey, conducted in 2014–15 following the UCC campaign, found that 90% of households owned at least one LLIN, while 69% of residents had slept under an LLIN the previous night [3]. However, more recent data indicate that Uganda suffers from a LLIN ownership gap of over 40%, representing the proportion of households that own at least one net, but not enough nets for every two occupants [1]. Furthermore, results from a comprehensive surveillance programme suggest that although the UCC successfully increased LLIN coverage levels in Uganda, the effect on clinical malaria indicators was limited [4]. These findings raise concerns about attrition and use of LLINs [12, 13], and the potential impact of pyrethroid resistance [4, 14, 15]. To better understand patterns of LLIN ownership, coverage, and use, a cross-sectional community survey in 48 districts in Eastern and Western Uganda was conducted. This is the first large-scale survey of LLIN coverage in Uganda since the 2014–15 Malaria Indicator Survey, and will serve as the baseline for an ongoing cluster-randomized trial to evaluate the impact of LLINs with, and without, piperonyl butoxide (PBO) on parasite prevalence in community children aged 2–10 years (ISRCTN 17516395).

## Methods

### Study design

This was a cross-sectional community survey conducted in 104 health sub-districts covering 48 of the 121 districts of Uganda. A household questionnaire was administered to 50 randomly selected households in each health sub-district.

### Study area

**Fig. 1:**
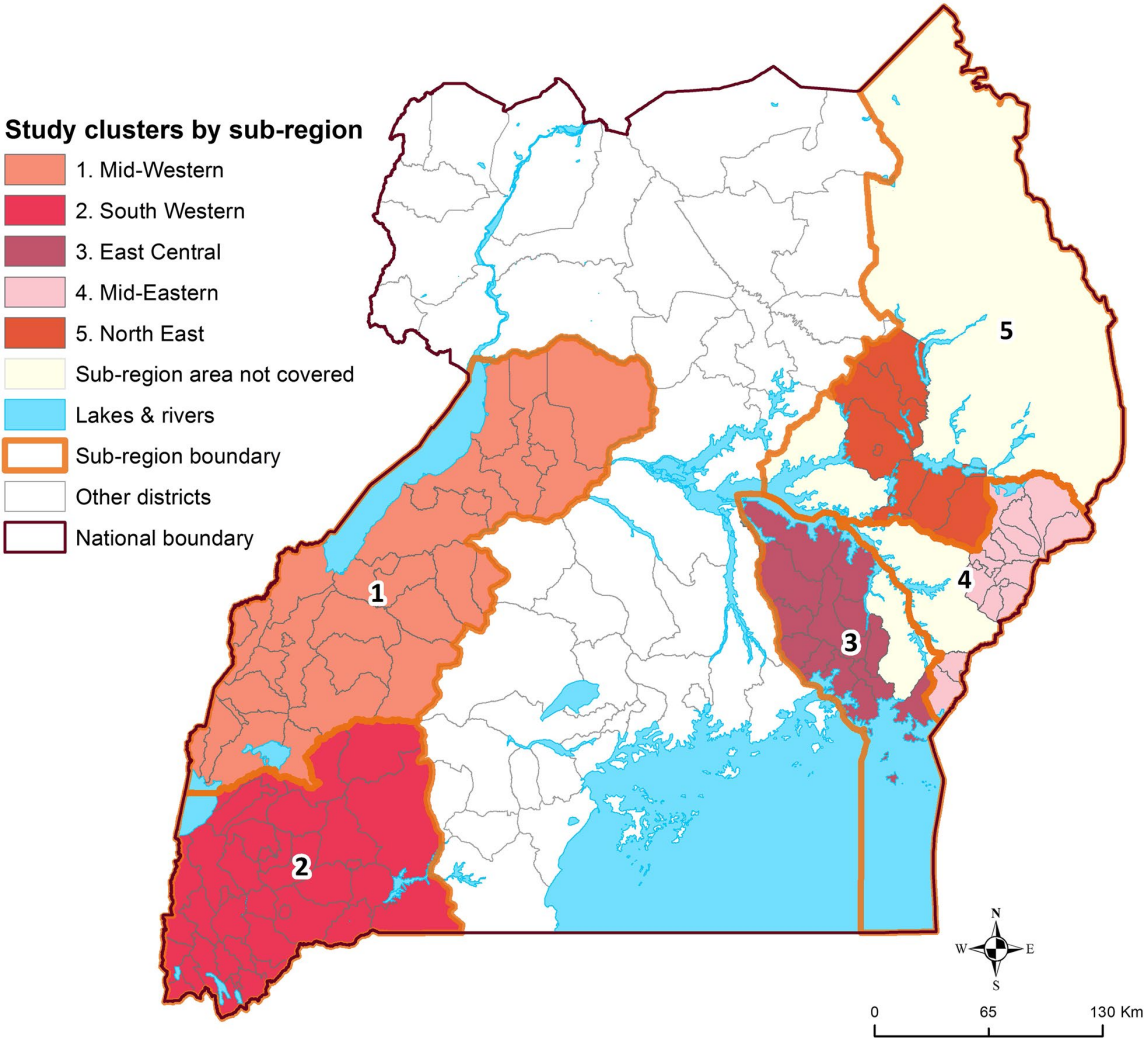
Map of the study area

The study area included 38 health sub-districts in the Eastern region and 66 in the Western region, covering 5 of the 10 administrative districts from the 2014–15 Malaria Indicator Survey (Fig. 1) [3]. This area represents approximately half of Uganda, with varying levels of malaria transmission and insecticide resistance [3, 4]. Health sub-districts scheduled to receive indoor residual spraying of insecticide (IRS) with pirimiphos-methyl (Actellic) were excluded due to an interim WHO recommendation, since retracted, that PBO nets should not be used in areas of Actellic spraying due to the possibility of antagonistic effects [16].

### Sampling frame

A two-stage cluster sampling procedure was applied, using enumeration areas identified in the 2014 national census as the primary sampling unit [3, 17]. Ten enumeration areas within each of the 104 health sub-districts were randomly selected by the Uganda Bureau of Statistics using probability proportionate-to-size sampling. Households within each selected enumeration area were mapped and enumerated by the study team. A random sample of mapped households was selected from each enumeration area to generate a list of households to approach for recruitment.

### Recruitment and enrolment

Households from the recruitment list were approached until 5 households from each enumeration area were enrolled (50 households per health sub-district, 5200 total). When a household was identified, study personnel briefly described the purpose of the study to the head of the household (or their designate) in the appropriate language. Households were included if: (1) at least one resident of the household was between 2 and 10 years of age, (2) at least one adult aged 18 years or older was present, (3) the adult was a usual resident who slept in the sampled household on the night before the survey, and (4) the adult resident agreed to provide informed consent for the household survey. Households were excluded if: (1) the dwelling was destroyed or not found, (2) the household was vacant, (3) there was no adult resident home on more than 3 occasions. Written consent to participate in the study was sought from an adult resident for all households fulfilling the selection criteria.

### Study procedures

Upon enrolment, a household survey questionnaire, adapted from prior cross-sectional community surveys conducted in Uganda including the national Malaria Indicator Survey [3, 18–20], was administered to heads of households or their designate using a hand-held tablet computer. Information was gathered on (1) the characteristics of households and residents, (2) proxy indicators of wealth including ownership of assets, and (3) ownership and use of LLINs in the households. The household survey was similar to the 2014 Malaria Indicator Survey and included the same questions in the same order on household asset ownership, water, sanitation, housing materials, use of IRS, and ownership and use of LLINs.

### Data management and statistical analysis

Data were collected using hand-held computers which were programmed to include range checks and internal consistency checks. Data were transferred daily to a secure server on a private network at the data core facility in Kampala. All statistical analyses were carried out using STATA version 15 (Statcorp, College Station, TX, USA). Three outcome measures were assessed, (1) LLIN ownership (the proportion of households that own at least one LLIN), (2) adequate LLIN coverage (the proportion of households that own at least one LLIN for every 2 occupants), and (3) LLIN use (the proportion of household residents who slept under an LLIN the previous night). Comparisons of LLIN ownership and coverage between the 2014–15 Malaria Indicator Survey and the 2017 survey conducted for this report only included data from health sub-districts included in both surveys. For each household included, the total number of LLINs was divided by the total number of residents, households with greater than or equal to 0.50 coverage were determined to have adequate nets. Categories of months since last UCC was based on the distribution of the data and relationships with the outcomes of interest following visual inspection using locally weighted scatterplot smoothing. A household wealth index was created using principal component analysis of data based on household ownership of various durable goods and land, and household food security; the wealth index was categorized into terciles. House type was classified as traditional or modern. Houses were defined as ‘modern’ if they had cement or wood or metal walls, a tiled or metal roof, and closed eaves. All other houses were classified as ‘traditional’ [21, 22]. Missing observations (n = 37) for variables included in the wealth index were assigned the mean value. Associations between predictors of interest and household-level outcomes (LLIN ownership and adequate coverage) were made using multivariate logistic regression. Associations between predictors of interest and individual-level outcomes (LLIN use) were made using multivariate generalized estimating equations with adjustment for clustering of study participants within the same household. The region of the country in which a household was located was included in the univariate analysis, but was excluded from the multivariate analysis due to collinearity with time since the last UCC.

## Results

### Characteristics of households, residents, and bed nets

**Fig. 2:**
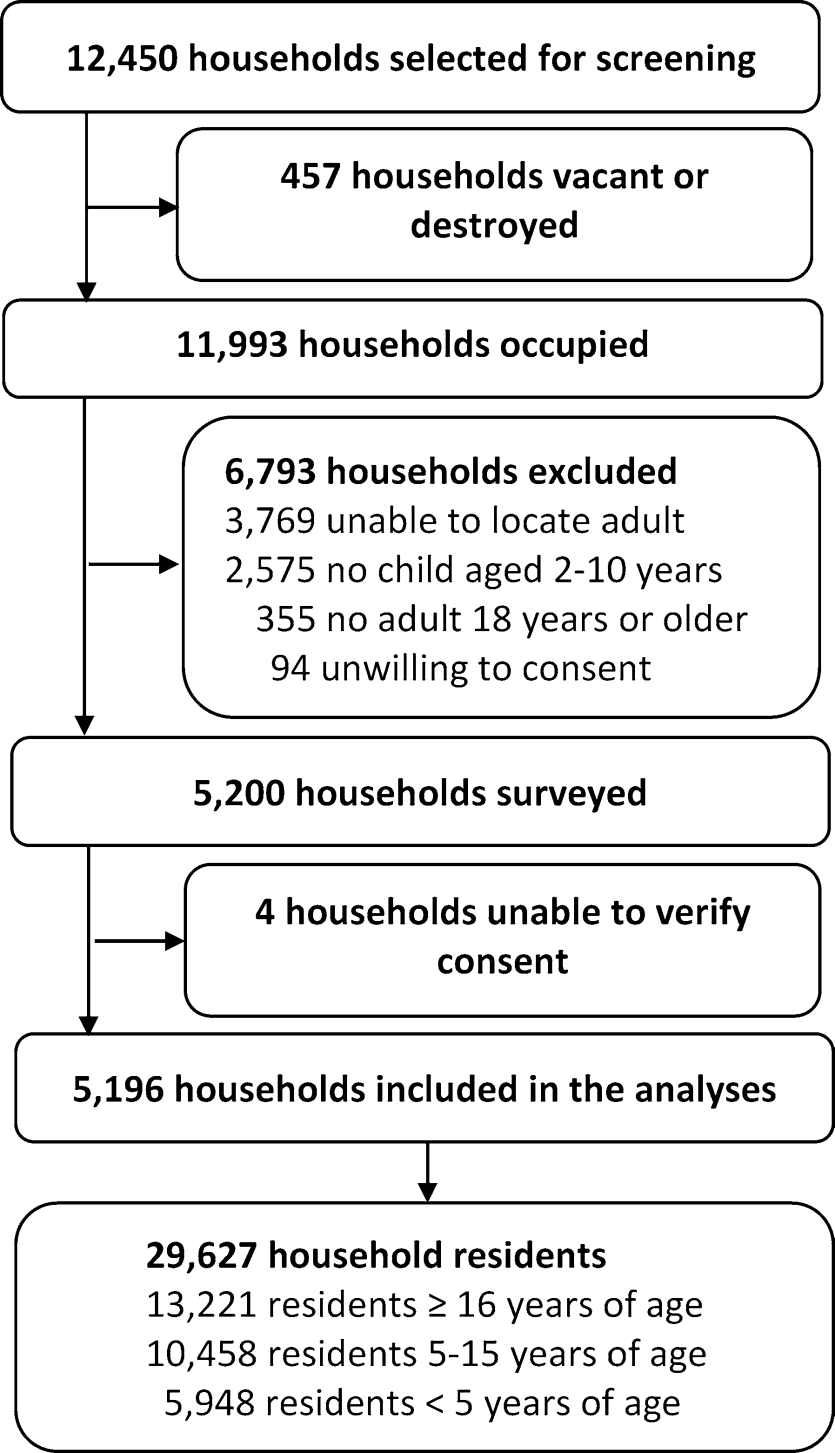
Trial profile

From March to June 2017, 5200 households were enrolled in the survey, and 5196 were included in the analysis (Fig. 2). Households received LLINs during the last national UCC 29–53 months prior to conducting the survey (Table 1). Most households were of traditional design, had at least one child aged less than 5 years, and were led by a male head of household (> 70% for all indicators). Of the 29,627 residents living in participating households, one-third were 5–15 years of age. Most, but not all, residents were a first-degree relative of the head of household. Only 14.8% of residents lived in a household with adequate LLIN coverage (at least one LLIN for every two occupants). Information was captured on 6980 bed nets; most nets had been obtained free-of-charge through a government campaign or from a public health facility, and approximately half of the nets were over 3 years old. Nearly all (93.5%) nets were LLINs; PermaNet 2.0 (Vestergaard, Lausanne, Switzerland) was the predominant brand.

**Table 1:** Characteristics of households, household residents, and bednets

Variable	Categories	n (%)
Characteristics at the level of the households (N = 5196)		
Region of the country	North East	350 (6.7%)
	Mid-East	750 (14.4%)
	East Central	800 (15.4%)
	Mid-Western	1448 (27.9%)
	South Western	1848 (35.6%)
Months since last Universal Coverage Campaign	29–37	3286 (63.2%)
	38–41	1260 (24.3%)
	42–53	650 (12.5%)
Wealth index	Poorest	1738 (33.5%)
	Middle	1727 (33.2%)
	Least poor	1731 (33.3%)
House type^a^	Traditional	3796 (73.1%)
	Modern	1400 (26.9%)
Estimated distance to nearest health facility	≥ 3 km	2441 (47.1%)
	< 3 km	2744 (52.9%)
Number of residents in household	2–4	1769 (34.1%)
	5–6	1872 (36.0%)
	≥ 7	1555 (29.9%)
Number of residents per sleeping space	≥ 3 residents	2382 (45.9%)
	< 3 residents	2813 (54.2%)
At least one resident < 5 years old	No	1254 (24.1%)
	Yes	3942 (75.9%)
Age of head of household (years)	< 30	907 (17.5%)
	30–39	1641 (31.6%)
	40–49	1280 (24.6%)
	≥ 50	1368 (26.3%)
Gender of head of household	Male	4041 (77.8%)
	Female	1155 (22.2%)
Characteristics at the level of the household residents (N = 29,627)		
Age in years	< 5	5948 (20.1%)
	5–15	10,458 (35.3%)
	> 15	13,221 (44.6%)
Gender	Male	14,029 (47.4%)
	Female	15,598 (52.7%)
Relationship to head of household	Head of household	5196 (17.5%)
	First degree relative	20,958 (70.7%)
	Second degree relative/unrelated	3473 (11.7%)
Lives in a household with adequate number of LLINs^b^	No	25,258 (85.3%)
	Yes	4369 (14.8%)
Characteristics at the level of each bednet identified (N = 6980)		
Where bednet was obtained	Government campaign	4207 (60.3%)
	Public health facility	1655 (23.7%)
	Private sector, church, friend/relative	1088 (15.6%)
	Unknown	30 (0.4%)
Whether bednet was purchased or not	Free	6129 (87.8%)
	Purchased	843 (12.1%)
	Unknown	8 (0.1%)
How long since bednet was obtained (months)	≤ 12	1144 (16.4%)
	13–24	1104 (15.8%)
	25–36	901 (12.9%)
	> 36	3759 (53.9%)
	Unknown	72 (1.0%)
Type of bednet	LLIN	6524 (93.5%)
	Untreated net	456 (6.5%)
Brand of LLIN	PermaNet 2.0	3598 (55.2%)
	Brand not specified	1109 (17.0%)
	Olyset net	636 (9.8%)
	DAWA net	525 (8.1%)
	PermaNet 3.0	196 (3.0%)
	Olyset plus	120 (1.8%)
	Other brand	119 (1.8%)
	Royal Sentry Net	117 (1.8%)
	Duranet	104 (1.6%)

^a^Modern houses were defined as those with a cement, wood or metal wall, tiled or metal roof and closed eaves; all other houses were defined as traditional

^b^At least one LLIN for every two people

### Changes in LLIN coverage over time

**Fig. 3:**
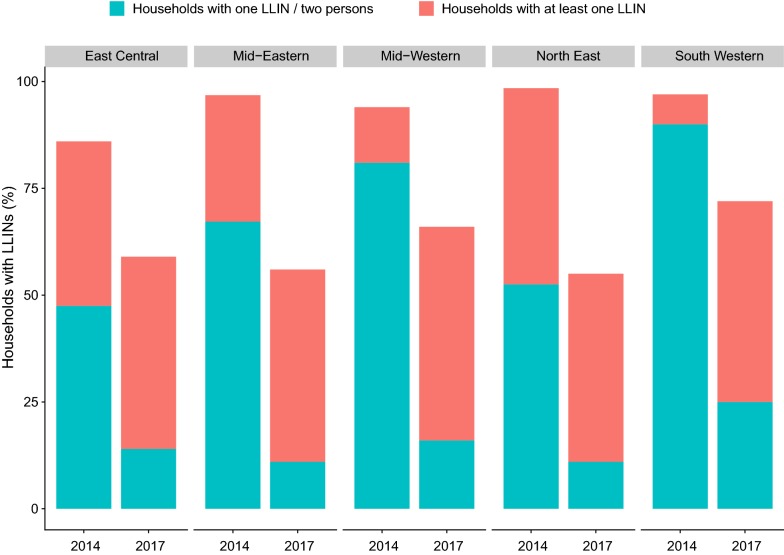
Change in LLIN ownership and coverage from 2014–15 to 2017

To assess changes in LLIN coverage over time, household ownership and adequate LLIN coverage measured in this survey, and in the 2014 Uganda Malaria Indicator Survey, were compared (Fig. 3). Overall, both household ownership and adequate LLIN coverage decreased substantially from 2014 to 2017; LLIN ownership decreased from 94 to 65% (p < 0.001), and adequate LLIN coverage decreased from 65 to 18% (p < 0.001). Regional differences in LLIN ownership over time were observed, with the greatest absolute decrease in ownership occurring in the North East (42%) and Mid-Eastern regions (39%). All regions experienced substantial absolute decreases in adequate LLIN coverage (ranging from 53% in the East Central to 70% in the North East).

### Factors associated with household ownership of at least one LLIN

Overall, 65.0% of households owned at least one LLIN; ownership varied by region and was highest in the South Western and Mid-Western regions (the regions with the shortest time since the last UCC). In the adjusted analysis, LLIN ownership was significantly associated with time since the last net campaign, household wealth, presence of a child under-five, and age and gender of the household head (Table 2). Household wealth and time since the last net campaign were the strongest predictors of household LLIN ownership; the odds of owning a LLIN were almost twice as high in the wealthiest households as compared to the poorest households (OR: 1.94, 95% CI 1.66–2.28, p < 0.001). Similarly, the odds of owning a LLIN were almost twice as high in households that had received nets more recently (29–37 months vs 42–53 months since the last net campaign).

**Table 2:** Factors associated with household ownership of at least one LLIN

Variable	Categories	Outcome present (%)	Univariate analysis		Multivariate analysis	
			OR (95% CI)	p-value	OR (95% CI)	p-value
Region of the country	Mid-North East	611 (55.6%)	Reference group		Not included due to strong correlation with months since last Universal Coverage Campaign distribution	
	East Central	471 (58.9%)	1.15 (0.95–1.38)	0.15		
	Mid-Western	959 (66.2%)	1.57 (1.34–1.84)	< 0.001		
	South Western	1338 (72.4%)	2.10 (1.80–2.46)	< 0.001		
Months since last Universal Coverage Campaign	42–53	338 (52.0%)	Reference group		Reference group	
	38–41	749 (59.4%)	1.35 (1.12–1.64)	0.002	1.28 (1.06–1.56)	0.01
	29–37	2292 (69.8%)	2.13 (1.79–2.53)	< 0.001	1.91 (1.60–2.28)	< 0.001
Wealth index	Poorest	973 (56.0%)	Reference group		Reference group	
	Middle	1138 (65.9%)	1.52 (1.32–1.74)	< 0.001	1.41 (1.23–1.63)	< 0.001
	Least poor	1268 (73.2%)	2.15 (1.87–2.48)	< 0.001	1.94 (1.66–2.28)	< 0.001
House type^a^	Traditional	2419 (63.7%)	Reference group		Reference group	
	Modern	960 (68.6%)	1.24 (1.09–1.42)	0.001	0.98 (0.85–1.14)	0.83
Estimated distance to nearest health facility	> 3 km	1554 (63.7%)	Reference group		Reference group	
	≤ 3 km	1820 (66.3%)	1.12 (1.00–1.26)	0.05	1.07 (0.95–1.20)	0.29
Number of residents in household	2–4	1096 (62.0%)	Reference group		Reference group	
	5–6	1250 (66.8%)	1.23 (1.08–1.41)	0.002	1.11 (0.96–1.28)	0.16
	≥ 7	1033 (66.4%)	1.22 (1.05–1.40)	0.007	1.01 (0.86–1.19)	0.87
Number of residents per sleeping space	≥ 3 people	1483 (62.3%)	Reference group		Reference group	
	< 3 people	1895 (67.4%)	1.25 (1.12–1.40)	< 0.001	1.10 (0.97–1.24)	0.15
At least one resident < 5 years old	No	791 (63.1%)	Reference group		Reference group	
	Yes	2588 (65.7%)	1.12 (0.98–1.28)	0.10	1.25 (1.08–1.44)	0.003
Age of head of household (years)	< 30	517 (57.0%)	Reference group		Reference group	
	30–39	1088 (66.3%)	1.48 (1.26–1.75)	< 0.001	1.35 (1.13–1.61)	0.001
	40–49	882 (68.9%)	1.67 (1.40–2.00)	< 0.001	1.56 (1.28–1.89)	< 0.001
	≥ 50	892 (65.2%)	1.41 (1.19–1.68)	< 0.001	1.42 (1.17–1.72)	< 0.001
Gender of head of household	Female	685 (59.3%)	Reference group		Reference group	
	Male	2694 (66.7%)	1.37 (1.20–1.57)	< 0.001	1.27 (1.10–1.47)	0.001

^a^Modern houses were defined as those with a cement, wood or metal wall, tiled or metal roof and closed eaves; all other houses were defined as traditional

### Factors associated with ownership of at least one LLIN for every two people

Overall, adequate coverage of LLINs was low; only 17.9% households reported owning at least one LLIN for every two people (Table 3). Adequate LLIN coverage also varied by region and was highest in the South Western and Mid-Western regions. In the adjusted analysis, adequate LLIN coverage was significantly associated with time since the last net campaign, household wealth, distance to the nearest health facility, number of residents in the household and per sleeping space, presence of a child under-five, and age of the head of household. The strongest predictor of adequate LLIN coverage was number of household residents with the odds of adequate coverage 6.5 times higher in smaller households (2–4 residents) than in the largest households (≥ 7 residents, OR: 6.50, 95% CI 5.11–8.26, p < 0.001). Household wealth and time since the last net campaign were also strong predictors of adequate LLIN coverage; the odds of adequate coverage were over twice as high in the wealthiest households as compared to the poorest households, and twice as high in households that had received nets more recently (29–37 months vs 42–53 months).

**Table 3:** Factors associated with household ownership of at least one LLIN for every two people

Variable	Categories	Outcome present (%)	Univariate analysis		Multivariate analysis	
			OR (95% CI)	p-value	OR (95% CI)	p-value
Region of the country	Mid-North East	116 (10.6%)	Reference group		Not included due to strong correlation with months since last Universal Coverage Campaign distribution	
	East Central	111 (13.9%)	1.37 (1.04–1.80)	0.03		
	Mid Western	232 (16.0%)	1.62 (1.28–2.05)	< 0.001		
	South Western	469 (25.4%)	2.88 (2.32–3.59)	< 0.001		
Months since last Universal Coverage Campaign	42–53	68 (10.5%)	Reference group		Reference group	
	38–41	161 (12.8%)	1.25 (0.93–1.69)	0.14	1.38 (1.01–1.89)	0.05
	29–37	699 (21.3%)	2.31 (1.77–3.01)	< 0.001	2.13 (1.61–2.81)	< 0.001
Wealth index	Poorest	211 (12.1%)	Reference group		Reference group	
	Middle	298 (17.3%)	1.51 (1.25–1.83)	< 0.001	1.52 (1.25–1.86)	< 0.001
	Least poor	419 (24.2%)	2.31 (1.93–2.77)	< 0.001	2.31 (1.88–2.84)	< 0.001
House type^a^	Traditional	625 (16.5%)	Reference group		Reference group	
	Modern	303 (21.6%)	1.40 (1.20–1.63)	< 0.001	1.04 (0.87–1.24)	0.67
Estimated distance to nearest health facility	> 3 km	366 (15.0%)	Reference group		Reference group	
	≤ 3 km	560 (20.4%)	1.45 (1.26–1.68)	< 0.001	1.33 (1.13–1.55)	< 0.001
Number of residents in household	≥ 7	111 (7.1%)	Reference group		Reference group	
	5–6	297 (15.9%)	2.45 (1.95–3.09)	< 0.001	2.67 (2.10–3.39)	< 0.001
	2–4	520 (29.4%)	5.42 (4.35–6.74)	< 0.001	6.50 (5.11–8.26)	< 0.001
Number of residents per sleeping space	≥ 3 people	286 (12.0%)	Reference group		Reference group	
	< 3 people	642 (22.8%)	2.17 (1.86–2.52)	< 0.001	1.57 (1.33–1.85)	< 0.001
At least one resident < 5 years old	Yes	617 (15.7%)	Reference group		Reference group	
	No	311 (24.8%)	1.78 (1.52–2.07)	< 0.001	1.22 (1.02–1.45)	0.03
Age of head of household (years)	< 30	151 (16.7%)	Reference group		Reference group	
	30–39	277 (16.9%)	1.02 (0.82–1.26)	0.88	1.23 (0.97–1.55)	0.08
	40–49	231 (18.1%)	1.10 (0.88–1.38)	0.40	1.66 (1.28–2.14)	< 0.001
	≥ 50	269 (19.7%)	1.23 (0.98–1.53)	0.07	1.61 (1.25–2.08)	< 0.001
Gender of head of household	Male	681 (16.9%)	Reference group		Reference group	
	Female	247 (21.4%)	1.34 (1.14–1.58)	< 0.001	1.02 (0.85–1.23)	0.80

^a^Modern houses were defined as those with a cement, wood or metal wall, tiled or metal roof and closed eaves; all other houses were defined as traditional

### Factors associated with LLIN use the previous night

Only 11,698 (39.5%) of residents reported sleeping under a LLIN the previous night (Table 4); use was lowest in the mid-north east and highest in the south-west. In an adjusted analysis, LLIN use the previous night was significantly associated with all factors included in the analysis. Time since the last net campaign, household wealth, relationship to the household head, and resident age were the strongest predictors of LLIN use; the odds of using LLINs were twice as high in residents of households that had received nets more recently, and almost twice as high in residents that lived in wealthier households as compared to the poorest households. Similarly, the odds of using LLINs were almost twice as high in residents that were head of the household (as compared to 2nd degree relatives or those unrelated to the household head), or were less than 5 years of age (as compared to children aged 5–15 years) (Table 4).

**Table 4:** Factors associated with household residents reporting sleeping under a LLIN the previous night

Variable	Categories	n (%)	Univariate analysis		Multivariate analysis	
			OR (95% CI)	p-value	OR (95% CI)	p-value
Region of the country	Mid-North East	2017 (31.1%)	Reference group		Not included due to strong correlation with months since last Universal Coverage Campaign distribution	
	East Central	1510 (32.3%)	1.06 (0.91–1.24)	0.45		
	Mid Western	3351 (40.0%)	1.48 (1.30–1.69)	< 0.001		
	South Western	4820 (47.9%)	2.09 (1.84–2.37)	< 0.001		
Months since last Universal Coverage Campaign	42–53	1036 (28.1%)	Reference group		Reference group	
	38–41	2509 (33.3%)	1.35 (1.14–1.59)	< 0.001	1.35 (1.14–1.60)	0.001
	29–37	8153 (44.3%)	2.14 (1.85–2.48)	< 0.001	2.02 (1.73–2.36)	< 0.001
Wealth index	Poorest	3032 (31.7%)	Reference group		Reference group	
	Middle	3873 (39.3%)	1.40 (1.25–1.57)	< 0.001	1.37 (1.22–1.53)	< 0.001
	Least poor	4793 (46.9%)	1.89 (1.70–2.11)	< 0.001	1.82 (1.62–2.04)	< 0.001
Estimated distance to nearest health facility	≥ 3 km	5315 (37.7%)	Reference group		Reference group	
	< 3 km	6369 (41.2%)	1.17 (1.07–1.28)	< 0.001	1.11 (1.01–1.22)	0.03
Number of residents per sleeping space	≥ 3 people	5195 (35.7%)	Reference group		Reference group	
	< 3 people	6500 (43.2%)	1.32 (1.21–1.44)	< 0.001	1.15 (1.04–1.26)	0.005
Number of household residents	≥ 7	4384 (33.2%)	Reference group		Reference group	
	5–6	4424 (43.5%)	1.51 (1.36–1.68)	< 0.001	1.43 (1.28–1.59)	< 0.001
	2–4	2890 (46.3%)	1.67 (1.49–1.87)	< 0.001	1.45 (1.29–1.63)	< 0.001
Households with at least one resident < 5 years old	No	2419 (37.2%)	Reference group		Reference group	
	Yes	9279 (40.1%)	1.17 (1.05–1.30)	0.003	1.20 (1.08–1.35)	0.001
Resident age in years	5–15 years	3209 (30.7%)	Reference group		Reference group	
	> 15 years	5832 (44.1%)	1.69 (1.61–1.77)	< 0.001	1.37 (1.29–1.45)	< 0.001
	< 5 years	2657 (44.7%)	1.67 (1.59–1.76)	< 0.001	1.71 (1.62–1.81)	< 0.001
Gender of resident	Male	5337 (38.0%)	reference group		reference group	
	Female	6361 (40.8%)	1.13 (1.10–1.17)	< 0.001	1.28 (1.23–1.34)	< 0.001
Relationship to head of household	2nd degree relative or unrelated	1083 (31.2%)	Reference group		Reference group	
	1st degree relative	8011 (38.2%)	1.19 (1.09–1.29)	< 0.001	1.14 (1.04–1.24)	0.005
	Head of household	2604 (50.1%)	1.81 (1.66–1.97)	< 0.001	1.82 (1.65–2.02)	< 0.001

### Factors associated with LLIN use with, and without, adequate coverage

Of the 29,627 residents, 4369 (14.8%) lived in a household with adequate LLIN coverage. The proportion of residents who slept under a LLIN the previous night was significantly higher in households with adequate LLIN coverage (89.0%) than in households without (30.9%). In households with adequate LLIN coverage, factors associated with LLIN use in an adjusted analysis included time since the last net campaign, presence of a child under-five, resident age and gender, and relationship to the household head (Table 5). The strongest predictors of LLIN use in households with adequate coverage were relationship to household head and time since the last net campaign; the odds of using LLINs were nearly 3.5 time higher if the resident was the head of household (as compared to 2nd degree relative or unrelated resident), and over twice as high in households that had received nets more recently.

**Table 5:** Comparison of members sleeping under an LLIN the previous night in households with and without adequate number of LLINs

Variable	Categories	Has adequate nets n (%)	Multivariate analysis		Does not have adequate nets n (%)	Multivariate analysis	
			OR (95% CI)	p-value		OR (95% CI)	p-value
Months since last Universal Coverage Campaign	42–53	241 (79.8%)	Reference group		795 (23.5%)	Reference group	
	38–41	721 (89.2%)	2.48 (1.34–4.61)	0.004	1788 (26.6%)	1.24 (1.04–1.49)	0.02
	29–37	2925 (89.8%)	2.31 (1.37–3.92)	0.002	5228 (34.5%)	1.76 (1.49–2.08)	< 0.001
Wealth index	Poorest	808 (89.0%)	Reference group		2224 (25.7%)	Reference group	
	Middle	1205 (87.9%)	0.95 (0.61–1.47)	0.81	2668 (31.5%)	1.30 (1.14–1.47)	< 0.001
	Least poor	1874 (89.7%)	1.11 (0.74–1.66)	0.62	2919 (35.9%)	1.55 (1.37–1.76)	< 0.001
Estimated distance to nearest health facility	< 3 km	1560 (90.6%)	Reference group		3755 (30.3%)	Reference group	
	≥ 3 km	2321 (88.0%)	0.73 (0.53–1.01)	0.05	4048 (31.6%)	1.05 (0.95–1.17)	0.33
Number of residents per sleeping space	≥ 3 people	1231 (88.0%)	Reference group		3964 (30.1%)	Reference group	
	< 3 people	2656 (89.4%)	1.30 (0.90–1.88)	0.16	3844 (31.8%)	0.97 (0.88–1.08)	0.63
Number of household residents	≥ 7	780 (87.2%)	Reference group		3604 (29.3%)	Reference group	
	5–6	1460 (89.5%)	1.27 (0.81–2.00)	0.30	2964 (34.7%)	1.19 (1.06–1.34)	0.002
	2–4	1647 (89.4%)	1.14 (0.73–1.77)	0.57	1243 (28.2%)	0.79 (0.69–0.91)	0.001
Households with at least one resident < 5 years old	No	1162 (84.3%)	Reference group		1257 (24.5%)	Reference group	
	Yes	2725 (91.1%)	2.15 (1.58–2.94)	< 0.001	6554 (32.5%)	1.44 (1.26–1.63)	< 0.001
Resident age in years	5–15 years	1217 (86.1%)	Reference group		1900 (37.1%)	Reference group	
	> 15 years	1913 (89.9%)	0.90 (0.71–1.14)	0.38	1992 (22.0%)	1.53 (1.42–1.64)	< 0.001
	< 5 years	757 (91.5%)	1.28 (1.00–1.65)	0.05	3919 (35.3%)	1.97 (1.84–2.11)	< 0.001
Gender of resident	Male	1781 (87.3%)	Reference group		3556 (29.7%)	Reference group	
	Female	2106 (90.5%)	1.52 (1.25–1.84)	< 0.001	4255 (32.1%)	1.34 (1.27–1.41)	< 0.001
Relationship to head of household	2nd degree relative or unrelated	454 (83.0%)	Reference group		1739 (40.8%)	Reference group	
	1st degree relative	2568 (88.7%)	1.50 (1.06–2.12)	0.02	5443 (30.1%)	1.22 (1.09–1.36)	< 0.001
	Head of household	865 (93.2%)	3.41 (2.02–5.29)	< 0.001	629 (21.5%)	2.12 (1.87–2.40)	< 0.001

In households without adequate LLIN coverage, factors associated with LLIN use in an adjusted analysis included time since the last net campaign, household wealth, number of residents per sleeping space and in the household, presence of a child under-five, resident age and gender, and relationship to the household head (Table 5). In households without adequate LLIN coverage, resident age and household wealth were strong predictors of LLIN use, in addition to time since the last net campaign and relationship to the household head. The odds of LLIN use were higher in wealthier households and in children under-five and residents > 15 years of age, compared to those aged 5–15 years.

## Discussion

LLINs are the cornerstone of vector control, but maintaining high coverage is a challenge. To better understand factors associated with LLIN ownership, coverage, and use, a cross-sectional survey in 48 districts across Uganda was conducted 29–53 months (2.4–4.4 years) after a national campaign aiming to achieve universal coverage. LLIN ownership, adequate coverage, and use of LLINs have all reduced markedly in Uganda since the 2014–15 Malaria Indicator Survey, suggesting that the Ugandan population is no longer adequately protected by LLINs. Time since the last UCC and household wealth were strongly associated with all outcome measures. Households that had received LLINs via the last UCC more recently, and wealthier households, were more likely to own and use LLINs, and to be adequately covered, as were smaller households with fewer residents. School-aged children were less likely to use nets, particularly in households without adequate coverage, as were residents who were more distantly related to the head of household. These findings highlight the important issue of net attrition. Strategies to minimize attrition and to target at-risk groups through continuous net distribution should be considered. National mass distribution campaigns may also need to be carried out more frequently.

To achieve and maintain universal LLIN coverage, the WHO recommends that countries distribute nets free-of-charge through universal coverage campaigns, supplemented by continuous distribution through different channels, including antenatal clinics [23]. LLINs are assumed to remain effective for at least 3 years under field conditions [24, 25], and the WHO recommends that mass campaigns be repeated every 3 years [10]. However, a growing body of evidence calls the 3-year lifespan of LLINs into question [26–30]. Although the WHO recognizes that ‘coverage gaps can start to appear almost immediately post-campaign due to net deterioration, loss of nets, and population growth’, the most recent guidelines on achieving and maintaining universal coverage with LLINs do not address strategies to minimize attrition [10]. Instead, emphasis is placed on the need to monitor national coverage and durability of LLINs [10]. Studies of net durability focus on survivorship (the proportion of nets distributed which remain in the household), physical integrity (presence of net damage, including the number and size of holes), and bio-efficacy (tested using WHO cone bioassays to assess mosquito knock-down and mortality after exposure). Survivorship of LLINs has been shown to range from 75% at 24 months after distribution in Rwanda [27], to 65% at 2–4 years of follow-up in Tanzania [28], to only 35% at 12 months post-distribution in Zambia [29]. Studies from Ethiopia and Madagascar suggest that physical deterioration of nets begins soon after distribution, with approximately half of LLINs assessed showing some damage by 6 months [26, 30]. In Ethiopia, 30% of nets were classified as unusable due to poor condition at 26–30 months [26], and in Tanzania, 39% of nets were considered unserviceable at 2–4 years [28]. In Zambia, only 56% of nets met criteria for functional survival (nets present and classified as ‘good’ or ‘damaged’) at 30 months giving an estimated mean survival time of 2.5–3 years [29], while in Rwanda, considering survivorship plus integrity, the lifespan of LLINs was estimated to be closer to 2 years [27]. Bio-efficacy results are mixed, but suggest that insecticidal effectiveness of LLINs may also be less than 3 years. In Tanzania, 63.6% of nets tested met the WHO cone assay criteria (caused more than 95% knock-down and/or > 80% mortality in pyrethroid susceptible *Anopheles gambiae* sensu stricto) 2–4 years after distribution [28], while in Zambia only 34.2% of LLINs met these criteria at 24 months (tested using susceptible *An. gambiae*) [29], and in Madagascar, average mortality was low at 12 months (ranging from 6.9 to 25.9% in susceptible *Anopheles arabiensis*) [30]. The net attrition documented in this study adds to this evidence, highlighting the need to re-evaluate the effective lifespan of LLINs under field conditions and to develop strategies to minimize attrition. Recognizing the operational challenges of delivering nets nationwide, the frequency of mass distribution campaigns and the approach to replacing nets through continuous distribution channels should also be reconsidered.

In this study, poorer households were less likely to own and use LLINs. The link between malaria and poverty is well-described [31–34], and multiple studies have demonstrated an association between poverty and poor LLIN coverage and use. Mass distribution of free LLINs has been strongly advocated to maximize coverage and ensure equitable access to LLINs [35]. However, evidence on whether distribution of free nets reduces inequity in protection by malaria control interventions, and risk of malaria infection, is limited. A recent study evaluated the change in equity in ownership of LLINs in 19 sub-Saharan African countries between 2003 and 2014, by comparing baseline and endline Demographic and Health Survey and Malaria Indicator Survey data. This study concluded that equity of net ownership had improved in 13 countries (including Uganda), was unchanged in 2 countries (Mali and Mozambique), and had worsened in 4 countries (favouring poorer households in Madagascar and Senegal; favouring wealthier households in Angola and Niger) [36]. Pooled multi-country analyses suggested that the significant increase in LLIN ownership favoured the poorest households in most countries. These results are encouraging, suggesting that mass distribution campaigns can reach poorer households. However, whether increased LLIN ownership translates into higher net use, and lower malaria risk, in poorer households is less clear. An analysis of Malaria Indictor Survey data collected in 2006–2009 in Angola, Tanzania and Uganda, aiming to evaluate the effectiveness of targeted distribution of free bed nets in achieving equity, found mixed results [37]. Wealth was strongly associated with household net ownership, as well as net use and malaria test positivity in children under-five. Distribution of free nets narrowed the gap in net ownership between poorer and wealthier households, but wealthier households were still more likely to own nets in all three countries, and use of nets by children in poorer households was not improved by distribution of free nets. In all three countries, children from poorer households were more likely to test positive for malaria than their wealthier counterparts [37]. These results suggest that household wealth is strongly associated with net ownership and use in Uganda, and that poorer households might be at risk for higher net attrition. Effective strategies to improve LLIN coverage and use among the poor, especially in between national distribution campaigns need to be identified.

In this study, school-aged children were less likely to use LLINs than children under-five or older residents. Typically, malaria control efforts have focused on children under-five (and pregnant women) because they bear the brunt of malaria morbidity and mortality. In endemic areas, risk of clinical disease and death declines throughout childhood due to the gradual acquisition of immunity gained through repeated infection [38]. By adolescence, most malaria infections are asymptomatic, although pregnancy again places women at increased risk. While older children generally experience fewer clinical malaria episodes in high transmission areas, the burden of malaria in schoolchildren is not insignificant, and has been associated with reduced school attendance, cognition, learning and school performance [39, 40]. Moreover, parasite prevalence is often highest in school-aged children, who serve as reservoirs of infection for the onward transmission of malaria [41–43]. Children in this ‘neglected’ age group are often less well-covered by LLINs [11, 42, 44], a finding supported by the results of this study. School-aged children suffer consequences of malaria and pose an important public health risk for transmission to other community members. This age group should be targeted with interventions aiming to improve LLIN coverage and use. School interventions are an attractive option given that schools are organized and well-distributed geographically, and thus provide access to a large proportion of this target group. Furthermore, children are considered change agents; targeting them can potentially lead to improved LLIN use within the household [45].

This study had several limitations. First, self-report was used to measure net use, which could have under-estimated or over-estimated the actual use of LLINs. Although nets were observed by field team members during the day-time surveys, night-time net use was not observed. Although this is the standard approach to measuring LLIN use [46], self-reported measures have been shown to overestimate LLIN adherence by 13.6% as compared to objective measures [47], suggesting that the true proportion of residents who slept under a LLIN the previous night could be lower than our estimates. Second, the ability to understand why individuals choose to use nets or not, is limited by the quantitative nature of the questionnaire. Further exploration using qualitative research methods would be required to better understand local perceptions and motivations for net use [12]. Third, although net attrition is an important issue, no data were collected on net quality or durability in this survey. However, as part of the LLINEUP project, net durability and bio-efficacy of LLINs will be assessed approximately 12 months after distribution via the 2017–18 national campaign in Uganda.

## Conclusions

In Uganda, LLIN ownership, coverage, and use were all well-below desired targets 2.5–4.5 years after LLINs were distributed through a national campaign. Net attrition may compromise the effectiveness of LLINs, leaving a substantial proportion of the population unprotected. The lifespan of LLINs under field conditions, and the optimum frequency of national LLIN mass distribution campaigns, should be reassessed. Moreover, strategies for continuous distribution of LLINs to schoolchildren and communities should be further explored to increase LLIN coverage of vulnerable groups. The results of this study will serve as a baseline for the ongoing Uganda PBO Net study, a cluster-randomised trial designed to evaluate the impact of LLINs, including conventional LLINs, and new generation LLINs combining a pyrethroid insecticide with a synergist (piperonyl butoxide), distributed by the Ugandan Ministry of Health in 2017–2018.

## Abbreviations

AMF: Against Malaria Foundation
IDRC: Infectious Diseases Research Collaboration
LLIN: long lasting insecticidal net
LLINEUP: long lasting insecticidal net Evaluation in Uganda Project
LSHTM: London School of Hygiene and Tropical Medicine
LSTM: Liverpool School of Tropical Medicine
MIS: Malaria Indicator Survey
MOH: Ministry of Health
NMCP: National Malaria Control Programme
PBO: piperonyl butoxide
UCSF: University of California San Francisco
UCC: Universal Coverage Campaign
UBOS: Uganda Bureau of Statistics
WHO: World Health Organization

## References

[1] WHO (2017). World malaria report 2017.

[2] Bhatt S, Weiss DJ, Cameron E, Bisanzio D, Mappin B, Dalrymple U (2015). The effect of malaria control on *Plasmodium falciparum* in Africa between 2000 and 2015. Nature.

[3] Uganda Bureau of Statistics (UBOS) and the National Malaria Control Programme of the Ugandan Ministry of Health (2015). Uganda malaria indicator survey 2014–15.

[4] Katureebe A, Zinszer K, Arinaitwe E, Rek J, Kakande E, Charland K (2016). Measures of malaria burden after long-lasting insecticidal net distribution and indoor residual spraying at three sites in Uganda: a prospective observational study. PLoS Med.

[5] Oguttu DW, Matovu JKB, Okumu DC, Ario AR, Okullo AE, Opigo J (2017). Rapid reduction of malaria following introduction of vector control interventions in Tororo District, Uganda: a descriptive study. Malar J.

[6] Raouf S, Mpimbaza A, Kigozi R, Sserwanga A, Rubahika D, Katamba H (2017). Resurgence of malaria following discontinuation of indoor residual spraying of insecticide in a previously high transmission intensity area of Uganda. Clin Infect Dis.

[7] Okullo AE, Matovu JKB, Ario AR, Opigo J, Wanzira H, Oguttu DW (2017). Malaria incidence among children less than 5 years during and after cessation of indoor residual spraying in Northern Uganda. Malar J.

[8] Lengeler C (2004). Insecticide-treated bed nets and curtains for preventing malaria. Cochrane Database Syst Rev.

[9] Kleinschmidt I, Bradley J, Knox TB, Mnzava AP, Kafy HT, Mbogo C (2018). Implications of insecticide resistance for malaria vector control with long-lasting insecticidal nets: a WHO-coordinated, prospective, international, observational cohort study. Lancet Infect Dis..

[10] WHO. Achieving and maintaining universal coverage with long-lasting insecticidal nets for malaria control. Geneva: World Health Organization, Global Malaria Programme; 2017. WHO/HTM/GMP/2017.20.

[11] Wanzira H, Katamba H, Rubahika D (2016). Use of long-lasting insecticide-treated bed nets in a population with universal coverage following a mass distribution campaign in Uganda. Malar J.

[12] Augustincic Polec L, Petkovic J, Welch V, Ueffing E, Tanjong Ghogomu E, Pardo Pardo J (2015). Strategies to increase the ownership and use of insecticide-treated bednets to prevent malaria. Cochrane Database Syst Rev..

[13] Strachan CE, Nuwa A, Muhangi D, Okui AP, Helinski ME, Tibenderana JK (2016). What drives the consistent use of long-lasting insecticidal nets over time? A multi-method qualitative study in mid-western Uganda. Malar J.

[14] Mawejje HD, Wilding CS, Rippon EJ, Hughes A, Weetman D, Donnelly MJ (2013). Insecticide resistance monitoring of field-collected *Anopheles gambiae* s.l. populations from Jinja, eastern Uganda, identifies high levels of pyrethroid resistance. Med Vet Entomol.

[15] Hemingway J, Ranson H, Magill A, Kolaczinski J, Fornadel C, Gimnig J (2016). Averting a malaria disaster: will insecticide resistance derail malaria control?. Lancet.

[16] WHO. Conditions for deployment of mosquito nets treated with a pyrethroid and piperonyl butoxide. Geneva: World Health Organization; 2017. WHO/HTM/GMP/2017.17.

[17] Steinhardt LC, Yeka A, Nasr S, Wiegand RE, Rubahika D, Sserwanga A (2013). The effect of indoor residual spraying on malaria and anemia in a high-transmission area of northern Uganda. Am J Trop Med Hyg.

[18] Uganda Bureau of Statistics (UBOS) and ICR Macro (2010). Uganda Malaria Indicator Survey 2009.

[19] Yeka A, Nankabirwa J, Mpimbaza A, Kigozi R, Arinaitwe E, Drakeley C (2015). Factors associated with malaria parasitemia, anemia and serological responses in a spectrum of epidemiological settings in Uganda. PLoS ONE.

[20] Staedke SG, Maiteki-Sebuguzi C, DiLiberto DD, Webb EL, Mugenyi L, Mbabazi E (2016). The impact of an intervention to improve malaria care in public health centers on health indicators of children in Tororo, Uganda (PRIME): a cluster-randomized trial. Am J Trop Med Hyg.

[21] Rek JC, Alegana V, Arinaitwe E, Cameron E, Kamya MR, Katureebe A (2018). Rapid improvements to rural Ugandan housing and their association with malaria from intense to reduced transmission: a cohort study. Lancet Planet Health..

[22] Wanzirah H, Tusting LS, Arinaitwe E, Katureebe A, Maxwell K, Rek J (2015). Mind the gap: house structure and the risk of malaria in Uganda. PLoS ONE.

[23] WHO (2014). Recommendations for achieving universal coverage with long-lasting insecticidal nets in malaria control (September 2013, revised March 2014).

[24] WHO. Guidelines for monitoring the durability of long-lasting insecticidal mosquito nets under operational conditions. Geneva: World Health Organization; 2011. WHO/HTM/NTD/WHOPES/2011.5.

[25] Kilian A, Byamukama W, Pigeon O, Gimnig J, Atieli F, Koekemoer L (2011). Evidence for a useful life of more than three years for a polyester-based long-lasting insecticidal mosquito net in Western Uganda. Malar J.

[26] Wills AB, Smith SC, Anshebo GY, Graves PM, Endeshaw T, Shargie EB (2013). Physical durability of PermaNet 2.0 long-lasting insecticidal nets over three to 32 months of use in Ethiopia. Malar J.

[27] Hakizimana E, Cyubahiro B, Rukundo A, Kabayiza A, Mutabazi A, Beach R (2014). Monitoring long-lasting insecticidal net (LLIN) durability to validate net serviceable life assumptions, in Rwanda. Malar J..

[28] Massue DJ, Moore SJ, Mageni ZD, Moore JD, Bradley J, Pigeon O (2016). Durability of Olyset campaign nets distributed between 2009 and 2011 in eight districts of Tanzania. Malar J..

[29] Tan KR, Coleman J, Smith B, Hamainza B, Katebe-Sakala C, Kean C (2016). A longitudinal study of the durability of long-lasting insecticidal nets in Zambia. Malar J..

[30] Randriamaherijaona S, Raharinjatovo J, Boyer S (2017). Durability monitoring of long-lasting insecticidal (mosquito) nets (LLINs) in Madagascar: physical integrity and insecticidal activity. Parasit Vectors..

[31] Gallup JL, Sachs JD (2001). The economic burden of malaria. Am J Trop Med Hyg.

[32] Sachs J, Malaney P (2002). The economic and social burden of malaria. Nature.

[33] Barat LM, Palmer N, Basu S, Worrall E, Hanson K, Mills A (2004). Do malaria control interventions reach the poor? A view through the equity lens. Am J Trop Med Hyg.

[34] Worrall E, Basu S, Hanson K (2005). Is malaria a disease of poverty? A review of the literature. Trop Med Int Health..

[35] Teklehaimanot A, Sachs JD, Curtis C (2007). Malaria control needs mass distribution of insecticidal bednets. Lancet.

[36] Taylor C, Florey L, Ye Y (2017). Equity trends in ownership of insecticide-treated nets in 19 sub-Saharan African countries. Bull World Health Organ.

[37] Njau JD, Stephenson R, Menon M, Kachur SP, McFarland DA (2013). Exploring the impact of targeted distribution of free bed nets on households bed net ownership, socio-economic disparities and childhood malaria infection rates: analysis of national malaria survey data from three sub-Saharan Africa countries. Malar J..

[38] Marsh K, Snow RW (1997). Host-parasite interaction and morbidity in malaria endemic areas. Philos Trans R Soc Lond B Biol Sci.

[39] Lalloo DG, Olukoya P, Olliaro P (2006). Malaria in adolescence: burden of disease, consequences, and opportunities for intervention. Lancet Infect Dis..

[40] Nankabirwa J, Brooker SJ, Clarke SE, Fernando D, Gitonga CW, Schellenberg D (2014). Malaria in school-age children in Africa: an increasingly important challenge. Trop Med Int Health..

[41] Stone W, Goncalves BP, Bousema T, Drakeley C (2015). Assessing the infectious reservoir of falciparum malaria: past and future. Trends Parasitol..

[42] Walldorf JA, Cohee LM, Coalson JE, Bauleni A, Nkanaunena K, Kapito-Tembo A (2015). School-age children are a reservoir of malaria infection in Malawi. PLoS ONE.

[43] Ouedraogo AL, Goncalves BP, Gneme A, Wenger EA, Guelbeogo MW, Ouedraogo A (2016). Dynamics of the human infectious reservoir for malaria determined by mosquito feeding assays and ultrasensitive malaria diagnosis in Burkina Faso. J Infect Dis.

[44] Pullan RL, Bukirwa H, Staedke SG, Snow RW, Brooker S (2010). Plasmodium infection and its risk factors in eastern Uganda. Malar J..

[45] Assefa M, Kumie A (2014). Assessment of factors influencing hygiene behaviour among school children in Mereb-Leke District, Northern Ethiopia: a cross-sectional study. BMC Public Health..

[46] MEASURE Evaluation. Household survey indicators for malaria control MEASURE DHS; 2013. https://data.unicef.org/resources/household-survey-indicators-for-malaria-control-2013-edition/.

[47] Krezanoski PJ, Bangsberg DR, Tsai AC (2018). Quantifying bias in measuring insecticide-treated bednet use: meta-analysis of self-reported vs objectively measured adherence. J Glob Health..

